# Remimazolam as an Adjunct to General Anesthesia in Children: Adverse Events and Outcomes in a Large Cohort of 418 Cases

**DOI:** 10.3390/jcm12123930

**Published:** 2023-06-08

**Authors:** Yoshitaka Kimoto, Tatsuya Hirano, Norifumi Kuratani, David Cavanaugh, Keira P. Mason

**Affiliations:** 1 Department of Anesthesiology, Kurume University School of Medicine, Kurume 830-0011, Japan; kimoto_yoshitaka@kurume-u.ac.jp; 2 Department of Anaesthesia, Saitama Children’s Medical Center, Saitama 330-8777, Japan; nori-kuratani@umin.ac.jp; 3 Department of Anaesthesia, National Hospital Organization Saitama Hospital, Saitama 351-0102, Japan; tatsuya.hirano721@gmail.com; 4 Boston Biostatistical Consulting, North Reading, MA 01864, USA; dmcav6@gmail.com; 5 Department of Anaesthesia, Critical Care and Pain Medicine, Boston Children’s Hospital, Boston, MA 02115, USA

**Keywords:** anesthesia, pediatrics, remimazolam

## Abstract

Remimazolam was first approved in 2020 as a general anesthetic for adults and still does not have pediatric labeling. Our study will be the first pilot program that administers remimazolam as an adjunct to a general endotracheal anesthetic in children. Between August 2020 and December 2022, electronic medical records were collected for all children who received remimazolam during anesthesia. The remimazolam dosing regimen was extrapolated from the adult package insert, with intravenous induction doses of 12 mg/kg/h administered until the desired effect was achieved. Subsequent infusions were given at a rate of 1–2 mg/kg/h, accompanied by intermittent boluses of 0.2 mg/kg, with all dosing adjustments made according to the anesthesiologist’s clinical discretion. A total of 418 children (mean 4.6 yrs, 68.7% ASA 1 and 2) underwent surgeries which averaged 81.2 min. A total of 75.2% of patients had greater than a 20% change (increase or decrease) in MAP (lowest or highest) from baseline, and 203 (49.3%) patients had greater than a 30% change (increase or decrease) in MAP (lowest or highest) from baseline. A total of 5% received ephedrine to treat unanticipated hemodynamic variability. Discharge criteria were met within an average of 13.8 min after arrival at the post-anesthesia care unit. Remimazolam may offer the benefits of rapid recovery following general endotracheal anesthesia. The risk of hemodynamic variability which necessitates and responds to ephedrine should be anticipated.

## 1. Introduction

Remimazolam is a soft drug of midazolam. It was first approved in Japan in January 2020 as a general anesthetic for adults and later approved in some countries of the European Union as well as Korea, also for general anesthesia. Metabolized by plasma tissue esterases to inactive metabolites, with a terminal half-life in adults of 0.92 h [1], remimazolam could offer advantages in the induction, maintenance, and recovery from general anesthesia [2].

Currently, there is no pediatric labeling for remimazolam, either for general anesthesia or sedation. A large multi-center Federal Drug Administration (FDA) pediatric trial to acquire labeling for sedation was initiated in April 2021 [3]. It is projected that pediatric approval is still years away. To date, there are no published pediatric studies that report a broad experience with remimazolam when administered as part of a general anesthetic. With only approval for adult administration, the off-label experience in the pediatric population has been limited to case reports only [4,5,6,7]. At our hospital, remimazolam is approved for off-label use as an adjunct to general anesthesia. The aim of this study is to provide the first comprehensive report on the outcomes of pediatric anesthesia with remimazolam, evaluating its safety and efficacy in a clinical setting. This will be the first report on the outcomes of a pediatric anesthesia program with remimazolam.

## 2. Materials and Methods

This study was approved by the Institutional Review Committee of Saitama Children’s Medical Center (Approval Code: 2022-05-025). Between August 2020 and December 2022, remimazolam was administered as an adjunct to general anesthesia at a tertiary care, academic pediatric hospital. Approved by their hospital’s Committee on Pharmaceutical Affairs for off-label pediatric application, remimazolam is administered as part of routine anesthesia clinical practice. With Institutional Review Board (IRB) approval, electronic medical records (EMR) from the peri-operative (pre, intra, and post-operative) periods were evaluated as an ongoing quality assurance (QA) initiative. Every child who received remimazolam as part of a general anesthetic during this time period was included in the study. Exclusion criteria comprised cases involving procedural sedation in the cardiac catheter laboratory and radiology department. The requirement for obtaining informed consent was waived by the Institutional Review Board, allowing for the analysis of patient information in this study. Remimazolam dosing was based on the approved adult labeling in Japan: Intravenous (IV) Induction doses of 12 mg/kg/h until the desired effect was achieved, with subsequent infusions of 1–2 mg/kg/h and intermittent boluses of 0.2 mg/kg at the anesthesiologist’s clinical discretion.

The primary outcome of this study was to identify the occurrence and incidence of severe critical events defined as an event requiring unplanned medical intervention with the potential for long-term disability, significant morbidity, or death [8,9]. The secondary outcomes examined the fluctuations in hemodynamics (heart rate and mean arterial blood pressure), emergence, and recovery characteristics.

Summaries of data were calculated using descriptive statistics: number of patients (n), mean, standard deviation (SD), median, minimum, and maximum, as well as frequency counts for categorical variables. Demographics and baseline characteristics: age, weight, body mass index, heart rate, MAP, and American Society of Anesthesiologists (ASA) score were summarized with descriptive statistics. Frequency counts of ASA score, type of procedure, and anesthesia induction method were also summarized. The occurrence of severe critical adverse events was identified using objective criteria applied in other multicenter, international anesthesia outcome trials: Laryngospasm, Bronchospasm, Pulmonary aspiration, Drug error, Anaphylaxis, Cardiovascular instability requiring unplanned intervention, Neurological damage, Perianaesthetic cardiac arrest, and Postanaesthetic Stridor. The occurrence of any of these events was considered a severe adverse event [8].

Hemodynamic fluctuations were evaluated by comparing each patient’s baseline set of vital signs to their highest and lowest recorded heart rate (HR) and mean arterial blood pressure (MAP) during the anesthetic. A deviation of 20% and 30% from each patient’s baseline (increase or decrease) MAP and heart rate (HR) was summarized with frequency counts. Other secondary clinical outcomes and parameters were summarized using descriptive statistics: Duration of Procedure (incision to final suture), duration of remimazolam, remimazolam administered (mg/kg/total time of procedure), Time from last dose of remimazolam to spontaneous eye opening in response to verbal stimulation (awakening), and PACU time (time spent in PACU from arrival to meet discharge criteria). Amount (or usage) of adjuvant anesthetics, narcotics, and vasopressors administered was summarized with descriptive statistics for the drugs which were administered: propofol, sevoflurane, fentanyl, remifentanil, morphine, ketamine, flumazenil, ephedrine, dobutamine, and dopamine. Statistical analyses were performed using SAS^®^ statistical software (version 9.4, SAS^®^ Institute, Cary, NC, USA).

## 3. Results

Four hundred eighteen (418) cases were recorded, comprising patients with a mean (SD) age of 4.6 (4.52) years old. The majority of patients were ASA 1 (30.4%) and ASA 2 (38.3%). There was a wide distribution of surgical procedures, with 83 (19.9%) classified as general surgery. Subjects had a similar distribution of intravenous (44.8%) versus inhalation (49.3%) anesthesia inductions. All patients were intubated for the anesthetic and surgical procedure. Demographics and baseline characteristics can be seen in Table 1.

A total of 21 (5.0%) patients received ephedrine to treat the heart rate and/or blood pressure, meeting the criteria of a severe adverse event as they received unplanned medical interventions for hemodynamic instability. Both patients who received dopamine and dobutamine were already receiving these vasopressors prior to transfer from the intensive care unit to the operating room and were continued on them as part of their pre-anesthetic plan. The use of these vasopressors was not a result or consequence of their anesthetic and was not identified as an intervention nor as an anesthesia-attributed adverse event.

Post-operative nausea and vomiting (PONV) occurred in 16 (13.8%) patients and were the only other adverse events documented. PONV occurred in the PACU and all were treated with and resolved after metoclopramide (the standard treatment at our institution). Flumazenil was administered to 55 (13.16%) children to hasten awakening (not for respiratory depression), a practice routinely employed at our institution. Flumazenil administration was not considered an adverse event.

Percent change from each patient’s pre-operatively documented baseline MAP and HR were calculated, examining the percentage deviation of the highest and lowest values from each individual’s baseline.

**Figure 1:**
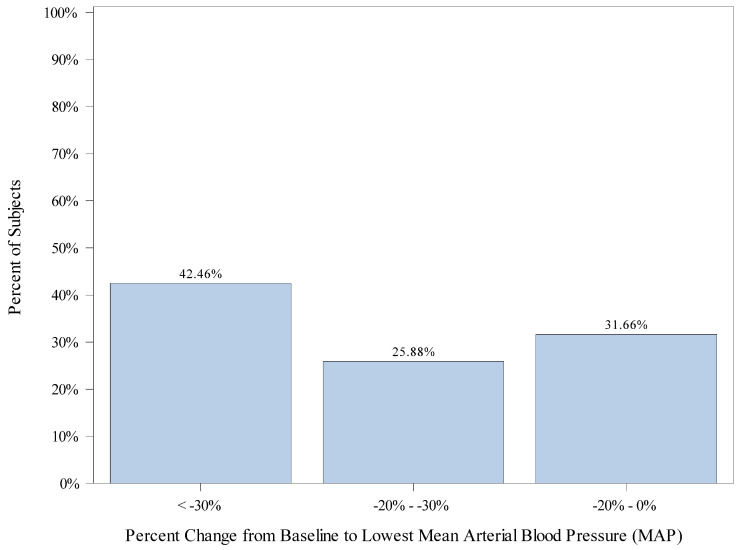
Distribution of Patients’ Change from Baseline to Lowest Mean Arterial Pressure (MAP).

The mean (SD) percent change from baseline to lowest MAP is −17.8 (12.4). A total of 398 (96.6%) subjects exhibited a decrease in MAP below their baseline. A total of 272 (68.3%) subjects had a percent change from baseline to lowest MAP of greater than −20%, including 103 (25.9%) subjects with a percent change from baseline greater than −20% but less than −30%, and 169 (42.5%) subjects with a percent change from baseline greater than −30%. A total of 126 (31.7%) children exhibited maximum drops in MAP that were within 20% of baseline. Figure 1 displays the percentage of subjects falling into categories of percent change from baseline to lowest MAP.

**Figure 2:**
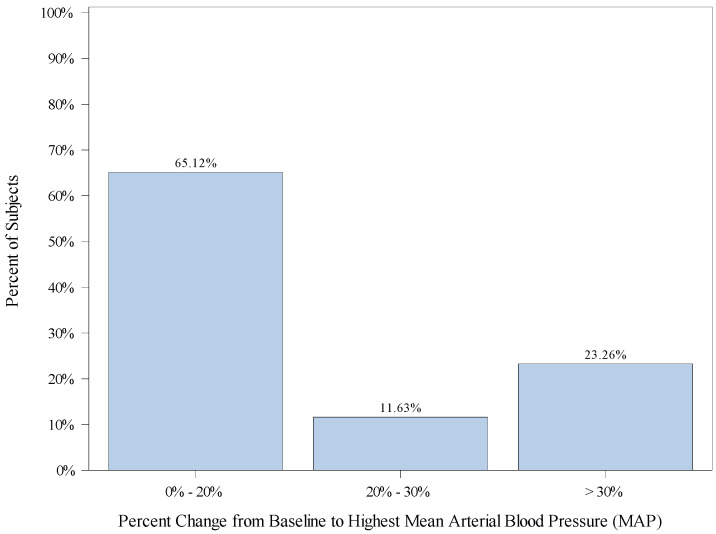
Distribution of Patients’ Change from Baseline to Highest Mean Arterial Pressure (MAP).

The mean (SD) percent change from baseline to highest MAP is 3.2 (16.13). A total of 240 subjects (58.3%) did not have a MAP that exceeded their baseline. A total of 60 (34.9%) subjects had a percent change from baseline to highest MAP of greater than 20%, including 20 (11.6%) subjects with a percent change greater than 20% but less than 30%, and 40 (23.3%) subjects with a percent change from baseline to highest MAP of greater than 30%. A total of 112 (65.1%) children had an elevation of MAP which was within 20% of baseline. Figure 2 displays the percentage of subjects falling into categories of percent change from baseline to highest MAP.

Overall, 310 (75.2%) patients had greater than a 20% change (increase or decrease) in MAP (lowest or highest) from baseline, and 203 (49.3%) patients had greater than a 30% change (increase or decrease) in MAP (lowest or highest) from baseline.

**Figure 3:**
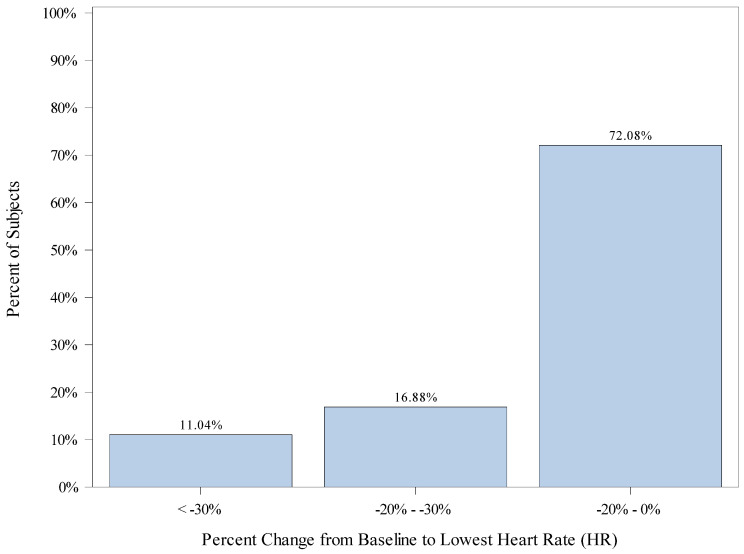
Distribution of Patients’ Change from Baseline to Lowest Heart Rate (HR).

Percent change from baseline was similarly compared to each patient’s lowest HR and highest HR. The mean (SD) percent change from baseline to lowest HR is −9.5 (19.20). A total of 109 subjects (26.1%) did not drop their heart rate below their recorded baseline. A total of 86 (27.0%) subjects had a percent change from baseline to lowest HR of greater than −20%, including 52 (16.9%) subjects with a percent change from baseline greater than −20% but less than −30%, and 34 (11.0%) subjects with a percent change from baseline greater than −30%. A total of 222 (72.1%) children had drops in heart rate that were within 20% of their baseline. Figure 3 displays the percentage of subjects falling into categories of percent change from baseline for the lowest HR.

**Figure 4:**
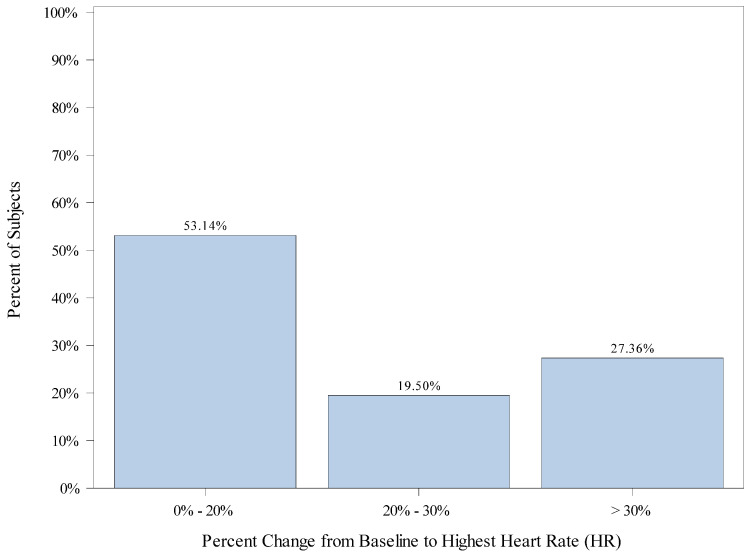
Distribution of Patients’ Change from Baseline to Highest Heart Rate (HR).

The mean (SD) percent change from each patient’s baseline to highest HR is 14.7 (23.36). There were 318 (76.3%) subjects that had an increase in highest HR and 99 (23.7%) subjects that did not have an increase in highest HR. A total of 149 (46.9%) subjects had a percent change from baseline to highest HR of greater than 20%, including 62 (19.5%) subjects with a percent change from baseline greater than 20% but less than 30%, and 87 (27.4%) subjects with a percent change from baseline greater than 30%. There were 169 (53.1%) subjects with a percent change from baseline to highest HR between 0% and 20%. Figure 4 displays the percentage of subjects falling into categories of percent change from baseline for highest HR.

Overall, 221 (53.0%) patients had greater than a 20% change (increase or decrease) in HR from baseline (lowest or highest), and 116 (27.8%) patients had greater than a 30% change (increase or decrease) in HR from baseline (lowest or highest).

The mean (SD) duration of the procedure (minutes) was 81.2 (75.58) and the mean (SD) duration (minutes) of remimazolam administration was 109.4 (83.1). The duration of anesthesia (minutes from the time of intubation to extubation) mean (SD) was 135.0 (88.36). The mean (SD) remimazolam administered (mg/kg/total time of procedure) was 0.48 (0.601). A total of 21 (5.0%) patients remained intubated at the end of the procedure, a decision that was not a result of an adverse event, and were transferred directly to the intensive care unit. Of all patients who were extubated, their mean (SD) time from discontinuation of remimazolam to “awakening” (spontaneous opening of eyes to verbal stimulation) was 34.3 (15.8) min. Comparatively, patients who received flumazenil had a mean (SD) time of awakening after discontinuing remimazolam of 34.7 (11.07) min. Those who did not receive flumazenil had a mean (SD) awakening time of 34.2 (15.5) min with a range of 12.0–108.0. There was no difference in awakening time between those groups that did and did not receive flumazenil (*p*-value 0.8282).

These patients who were extubated in the operating room met PACU discharge criteria on average 13.8 min (range 1–40) after arrival in PACU. Comparatively, those who received flumazenil met PACU discharge criteria mean (SD) in 15.5 (8.2) min, range 6.0–40.0 min. Those who did not receive flumazenil met PACU discharge criteria mean (SD) of 13.5 (6.9) min, range 1.0–31.0. There was no difference in time to meet discharge criteria between those who did and did not receive flumazenil (*p*-value 0.2565). Summary results of the procedure, perioperative, and recovery characteristics are summarized in Table 2. Table 3 displays the summary statistics for adjunct medications administration during the anesthetic: Remifentanil, fentanyl, and sevoflurane were the most common. Table 4 displays the PACU discharge criteria.

## 4. Discussion

Since remimazolam was first approved in 2020 in Japan for adults for general anesthesia, subsequent approvals for both sedation and anesthesia have varied based on the country. Approvals have varied with respect to applications (general anesthesia versus sedation), dosing, and method of administration (bolus versus infusion). Remimazolam is approved for general anesthesia in Japan, South Korea, and the European Union. The dosing for general anesthesia differs between those countries that have approval, ranging from an anesthesia infusion rate of no more than 12 mg/kg/h for induction, with a maintenance infusion of 1–2 mg/kg/h [10,11,12,13,14,15].

Without pediatric approval, the off-label use of remimazolam for general anesthesia has been limited to published accounts of case reports and small series [4,5,6,7,16]. At our institution, remimazolam has been approved for routine pediatric anesthesia use since 2020. For general anesthesia, remimazolam has been used as an adjunct, in conjunction with other anesthetics or opiates: remifentanil, fentanyl, and sevoflurane being the most common. In our experience, the recovery profile after discontinuing remimazolam has been rapid, both to meeting discharge criteria upon arrival to the post-anesthesia care unit and to the spontaneous opening of eyes in response to verbal stimulation.

Our experience reports that patients awakened (spontaneous eye movement) in an average of 34 min after discontinuing the remimazolam, longer than the reports of 19 min average to return to full consciousness after discontinuing infusions in adults [17]. This difference could be attributed to the fact that we are only reporting eye opening to verbal responses and that our patients were intubated and under general anesthesia, in contrast to the adults who were receiving only remimazolam for sedation purposes. On average, the children spent 13.8 min in PACU before they met discharge criteria. Although this rapid recovery cannot be definitively attributed to remimazolam, its rapid terminal half-life of 70 min (in adults) could be contributing to this rapid recovery [17]. There was no difference in awakening or PACU discharge eligibility in those patients who received flumazenil. As the administration of flumazenil was at the anesthesiologist’s discretion, future studies could set parameters for flumazenil administration in order to determine whether there is a benefit to the recovery profile.

Our observed rate of PONV was 13%, lower than the adult studies which reported a 35% incidence when remimazolam was administered as a total intravenous anesthetic [18]. Future studies are needed to determine whether remimazolam has a synergistic effect with some of the medications that are delivered during a general anesthetic and its individual effect on the recovery.

We observed a wide range of hemodynamic variability during our anesthetics. A total of 75.2% of the patients had greater than a 20% change (increase or decrease) in MAP (lowest or highest) from baseline, and 49.3% had greater than a 30% change (increase or decrease) in MAP (lowest or highest) from baseline. It is important to recognize that 31.7% and 72.1% exhibited drops in MAP and HR, respectively, that were within 20% of their baseline. A total of 26% of the children never dropped their heart rate below their recorded baseline. Variability in heart rate and blood pressure have been established to occur with remimazolam in adult trials. In the early adult trials, MAP decreased by 24% and heart rate increased by 28% [17].

In adult trials that compare propofol to remimazolam for induction and maintenance, the incidence of hypotension with remimazolam was dose-dependent, ranging from 20 to 24% compared to a 49% incidence with propofol [19]. Comparing the hemodynamic response to propofol and remimazolam during induction in adults, only remimazolam was determined to balance the sympathetic and parasympathetic response [20]. Comparing the high (12 mg/kg/h) and low (6 mg/kg/h) remimazolam induction doses, the efficacy and adverse event profile have not been shown to differ, even in the high-risk ASA 3 adults [21]. When compared to etomidate for adults undergoing cardiac surgery, low-dose remimazolam conferred similar hemodynamic stability [22]. Our patients received predominantly sevoflurane, remifentanil, and fentanyl. It will be important to determine in future studies, whether there is a synergy and the combined and individual effect of remimazolam on hemodynamics.

The incidence of significant adverse events was 5%, all attributed to the need for ephedrine to treat unanticipated hemodynamic fluctuations in mean arterial blood pressure or heart rate. The MAP and heart rates responded to the ephedrine and there were no subsequent hemodynamic variability or sequela noted intra-operatively nor in the post-anesthesia care unit. Our 5% incidence of cardiovascular instability, however, is high compared to the 1.9% cited in a large, prospective, multicenter international pediatric anesthesia observational study of over 30,000 children in 261 European hospitals [8]. None of the patients in this large study, however, received remimazolam. Larger, multicenter studies are needed to determine whether there is an association between remimazolam and hemodynamic variability or whether the adverse events that we identified are unrelated to the remimazolam.

This study, a retrospective case–series review, bears inherent limitations that ought to be addressed. Chiefly, the use of remimazolam was largely predicated on the discretion of the attending anesthesiologists, thus raising potential bias and variability in its administration. Remimazolam dosing adhered to the guidelines stipulated for adult general anesthesia in Japan, yet this did not eliminate the possibility of disparate decision-making across cases. While adverse events and outcomes did occur during the anesthetic period, their association with remimazolam is not unequivocally established. Notably, remimazolam was often employed as an adjunct to other anesthetics or opiates such as remifentanil, fentanyl, and sevoflurane, a practice that potentially accelerated recovery but simultaneously introduced confounding variables into the evaluation of remimazolam. The concurrent administration of other medications and clinical events, which may impact hemodynamic stability, represent further confounding factors. A more controlled study design could effectively isolate the effects of remimazolam, and our findings underscore the need for such rigorous investigation. Our observations on recovery times were indeed influenced by the concomitant administration of benzodiazepine antagonists, emphasizing the need for methodical, predefined administration parameters for antagonists such as flumazenil in future studies. This would yield more reliable data on the direct effects of remimazolam on recovery periods. The Bispectral Index (BIS), despite its ubiquity, has notable limitations as a pharmacodynamic reference, particularly given the absence of age-related covariates. Furthermore, potential bias emerges from the simultaneous use of other GABAergic agents with remimazolam, which was necessitated by our institutional practices and previous experiences. We recognize that the pharmacokinetic characteristics of remimazolam, including its relatively small central volume of distribution, may predispose to overdosing during rapid infusions. Regrettably, the inability to measure plasma levels of remimazolam in this study constitutes a significant limitation. The treatment of hemodynamic changes, as experienced by 5% of our patients, relied on the administration of ephedrine, a decision made at the anesthesiologist’s discretion rather than dictated by a protocol or guidelines. The observed rate of significant adverse events necessitating ephedrine due to hemodynamic fluctuations may seem elevated, suggesting further investigation to distinguish between genuine associations with remimazolam, incidental occurrences, or factors extraneous to the drug itself. Lastly, as this study was a retrospective review relying on electronic medical records to identify outcomes, it is plausible that we missed capturing some physiological or surgical events and patient attributes that may have precipitated or contributed to adverse events. Future research would benefit from a prospective design that allows for comprehensive data capture and minimizes these limitations.

## 5. Conclusions

Children who receive remimazolam as an adjunct to general endotracheal anesthesia demonstrate hemodynamic variability which may in some cases warrant therapy with ephedrine. Patients who receive intraoperative remimazolam, exhibit a rapid return to responsiveness and ability to meet discharge criteria. Future large, multicenter trials are needed in order to determine how the contribution of remimazolam affects these outcomes and how these effects are altered when administered in conjunction with other anesthetics and opiates.

## Figures and Tables

**Table 1:** Demographics and Baseline Characteristics.

Variable	Statistics (*n* = 418)	
Age (yrs)	Mean (SD)	4.6 (4.52)
	Median	3.0
	Min, Max	0.0, 17.9
Weight (kg)	Mean (SD)	16.5 (13.50)
	Median	12.1
	Min, Max	2.1, 75.9
BMI	Mean (SD)	16.2 (2.92)
	Median	16.1
	Min, Max	6.9, 29.0
ASA Score	Mean (SD)	1.9 (0.77)
	Median	2.0
	Min, Max	1.0, 4.0
ASA Score, *n* (%)	1	127 (30.4)
	1E	21 (5.0)
	2	160 (38.3)
	2E	16 (3.8)
	3	78 (18.7)
	3E	13 (3.1)
	4	1 (0.2)
	4E	2 (0.5)
Baseline Heart Rate (bpm)	Mean (SD)	107.5 (20.68)
	Median	108.0
	Min, Max	50.0, 169.0
Baseline MAP (mmHg)	Mean (SD)	65.0 (9.25)
	Median	64.0
Types of Procedure (%)	Central venous catheter insertion	22 (5.3)
	Dental surgery	1 (0.2)
	Gastrointestinal endoscopy	65 (15.6)
	General surgery	83 (19.9)
	Neurosurgery	17 (4.1)
	Ophthalmic surgery	10 (2.4)
	Orthopedic surgery	40 (9.6)
	Otorhinolaryngological surgery	21 (5.0)
	Percutaneous biopsy	15 (3.6)
	Plastic surgery	62 (14.8)
	Spine surgery	11 (2.6)
	Thoracic surgery	4 (1.0)
	Urologic surgery	39 (9.3)
	Others	28 (6.7)
Anesthesia Induction Method, *n* (%)	Already intubated	12 (2.9)
	Intravenous (IV)	187 (44.8)
	Rapid Sequence (IV) Induction	13 (3.1)
	Inhalation	206 (49.3)

**Table 2:** Summary of Procedures and Time to Discharge/Awakening.

Variable	Statistics	
Duration of Procedure (mins)	Mean (SD)	81.2 (75.58)
	Median	55.0
	Min./Max.	3.0/490.0
Remimazolam administered (mg/kg/total time that reimimazolam administered)	Mean (SD)	0.48 (0.601)
	Median	0.29
	Min./Max.	0.01/4.09
Time from last dose of remimazolam to awakening * (mins)	Mean (SD)	34.3 (15.80)
	Median	31.0
	Min./Max.	12.0/108.0
PACU time ** (mins)	Mean (SD)	13.8 (7.08)
	Median	13.0
	Min./Max.	1.0/40.0

* spontaneous eye opening to verbal stimulation. ** from arrival in PACU to meet discharge criteria.

**Table 3:** Summary of Drug Administration.

Variable	Statistics (mg)	
Total Amount Propofol (*n* = 45)	Mean (SD)	79.1 (78.21)
	Median	50.0
Total Amount Sevoflurane (*n* = 230)		
Total Amount Fentanyl (*n* = 364)	Mean (SD)	0.1 (0.12)
	Median	0.1
Total Amount Remifentanil (*n* = 395)	Mean (SD)	1.5 (1.64)
	Median	1.0
Total Amount Morphine (*n* = 71)	Mean (SD)	3.0 (1.66)
	Median	3.0
Total Amount Ketamine (*n* = 12)	Mean (SD)	14.8 (9.53)
	Median	10.0
Total Amount Flumazenil (*n* = 55)	Mean (SD)	0.2 (0.16)
	Median	0.2
Total Amount Ephedrine (*n* = 21)	Mean (SD)	7.6 (5.14)
	Median	8.0
Total Amount Dobutamine (*n* = 1)	Mean (SD)	2.3 (NA)
Total Amount Dopamine (*n* = 1)	Mean (SD)	5.4 (NA)

**Table 4:** PACU Discharge Criteria.

Spontaneous eye opening in response to verbal stimulation or mild tactile stimulation
Absent, minimal, or mild pain/discomfort
Spontaneous movements of extremities to verbal request or in response to mild stimulation
Absent or mild nausea
Oxygen saturation ≥ 97%
Normal respiratory pattern
No extreme tachycardia or bradycardia
No extreme hypertension or hypotension
Absence of shivering
Surgical wounds and conditions acceptable per surgical service
Temperature ≥ 36 °C but afebrile

## Data Availability

Data sharing is not applicable to this article. This was approved by the Institutional Review Board of Saitama Children’s Medical Center.
